# Regulatory T Cells in Multiple Sclerosis Diagnostics—What Do We Know So Far?

**DOI:** 10.3390/jpm14010029

**Published:** 2023-12-25

**Authors:** Borros Arneth

**Affiliations:** 1 Institute of Laboratory Medicine and Pathobiochemistry, Philipps University Marburg, 35043 Marburg, Germany; borros.arneth@staff.uni-marburg.de; 2 Institute of Laboratory Medicine and Pathobiochemistry, Justus Liebig University Giessen, 35392 Giessen, Germany; 3 Hospital of the Universities of Giessen and Marburg, 35392 Giessen, Germany

**Keywords:** endocrine system, endocrinology, infections

## Abstract

Background: Multiple sclerosis (MS) is an autoimmune disorder that affects the central nervous system (CNS) through inflammation. MS symptoms become acute if the disease progresses to the relapsing phase. Aim: This review aimed to evaluate the role played by regulatory T cells (Tregs) in the pathogenesis of MS. Methods: This review used scholarly journal articles obtained from PubMed, PsycINFO, and CINAHL with different search parameters such as ‘regulatory T cells’, ‘multiple sclerosis’, and ‘current knowledge’. The process of searching for articles was limited to those that had publication dates falling between 2010 and 2020. Results: Tregs play a role in the pathogenesis of MS. This conclusion is supported by animal disease models and environmental factors that can underlie Treg alterations in MS. Despite the knowledge of the role played by Tregs in MS pathogenesis, the specific subsets of Tregs involved in MS development remain incompletely understood. Discussion: This review provides an essential link between Tregs and MS activity. Targeting Tregs could be an efficient way to establish new treatment methods for MS management. Conclusion: MS is a complex condition affecting many people worldwide. Research has shown that Tregs can influence MS development and progression. More investigations are needed to understand how Tregs affect the pathogenesis of MS.

## 1. Introduction

Multiple sclerosis is an autoimmune disease. In this disease, cells of the immune system target the body’s own structures, namely the oligodentrocytes—the coverings of the nerve cells. The regulatory T lymphocytes are believed to be involved in this dysregulation. For this reason, this review aims to clarify our up-to-date knowledge of whether the regulatory cells can help to better diagnose and/or treat multiple sclerosis.

The review examines how Treg cells influence multiple sclerosis. The study adhered to the Preferred Reporting Items for Systematic Reviews and Meta-Analyses (PRISMA) statement standards, providing a framework for searching and examining the relevant literature [1]. The study addresses the following PICO question: “In patients diagnosed with multiple sclerosis and animals under experimental autoimmune encephalomyelitis (EAE), does the use of regulatory T cells for diagnosis and/or therapy monitoring compared with standard treatment or control groups without specific modulation of regulatory T cells result in a measurable impact/decrease on disease progression, relapse rates, and/or improvement in clinical symptoms related to multiple sclerosis?”

The first step involved searching for relevant articles in scientific databases and libraries such as PubMed, EMBASE, Cochrane, and Google Scholar. The keyword combinations included:

(i) Regulatory T cells OR Treg cells OR T regulatory cells OR CD4+CD25+ regulatory T cells
(ii) Regulatory T cells or Treg cells + experimental autoimmune encephalomyelitis (EAE)
(iii) Multiple sclerosis OR MS OR central nervous system autoimmune disease
(iv) Regulatory T cells or Treg cells for diagnosing multiple sclerosis
(v) Regulatory T cells or Treg cells in therapy monitoring of multiple sclerosis.

### Criteria for Inclusion and Exclusion of Studies

The specificity of the PICO question necessitates strategic inclusion and exclusion criteria to find ideal journal articles. The use of regulatory T cells for diagnosing and in therapy monitoring of MS has been documented in vast studies, and the findings can reveal whether they affect relapse rates, disease progression, or improvements. The inclusion and exclusion criteria focus on the types of studies, types of participants, types of interventions, and time frame of the field trials. The subsequent paragraphs provide an in-depth perspective of the inclusion/exclusion criteria above.

The study focused on examining original research peer-reviewed journals for the review. The original research journals provide reliable information backed by empirical or evidence-based research to address an issue. Sürücü and Maslakci state that empirical researchers use quantitative measurements that augment the validity and reliability of the study [2]. The high validity and reliability levels minimize bias and offer a comprehensive understanding of the study phenomenon. Thus, the researcher will only select original journal articles to sustain the desired quality threshold.

The types of study participants comprise patients diagnosed with MS and Treg cell diagnosis or therapies used as interventions. Treg cells are disease markers in MS patients and protect healthy individuals from autoimmune disease. Verma et al. state that MS patients have a decreased resting and an increased activation of CD4+ and CD25+ T cells [3]. The T cells reveal diverse information about the MS progression and the efficacy of the various interventions. Examining studies with the prescribed subjects offers a detailed perspective of the role of Treg cells in MS management.

The types of interventions include therapies or evidence-based practices that regulate Treg cells to manage MS. The therapies reveal the impact of moderating the regulatory Treg cells in MS treatment. Islam et al. describe a few therapies that utilize Treg cell modulation to manage MS [4]. The evidence-based studies or therapies offer distinct results that exhibit the extent of knowledge in using Treg cells to manage MS. The therapies and evidence-based studies offer unique and multifaceted information, providing a wealth of knowledge.

The time frame of the research paper publication spans ten years. The research papers must have been published between 2013 and 2023, featuring new content. Recent studies provide building blocks for the study through peer-reviewed fresh data, ensuring quality and accuracy [5]. The integration of recent research ensures the integration of current information and diverse perspectives and addresses recent gaps in the study. The recent studies enable the researcher to integrate contemporary information and make conclusions based on recent findings.

**Figure 1:**
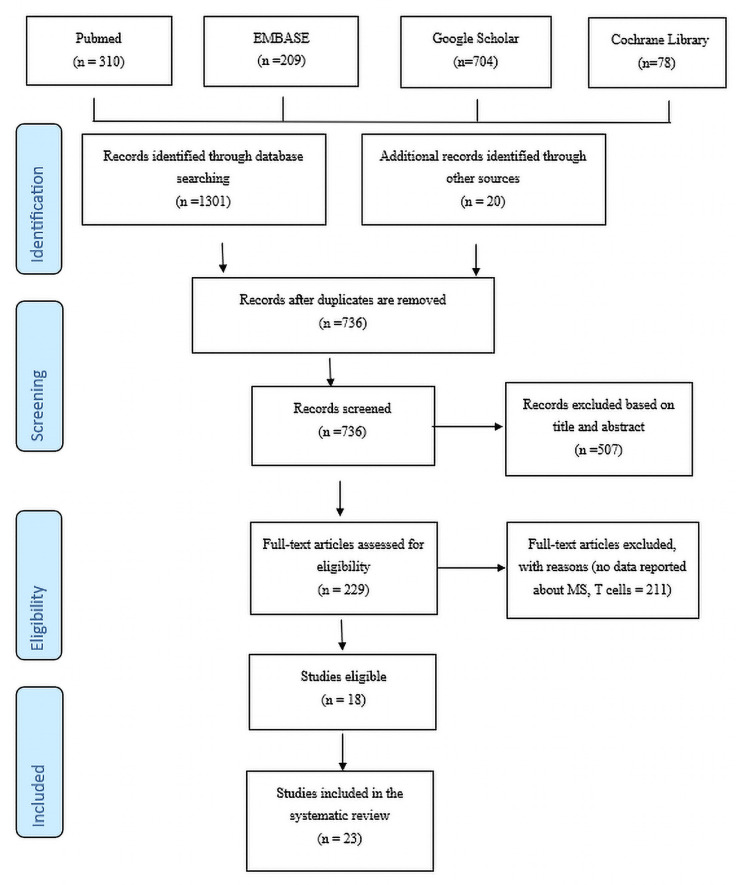
PRISMA flowchart.

The researcher will exclude non-English publications, meeting reports, or abstract forms from the quality assessment table. The content of the abstract will be examined to determine any indication that the research focused on the influence of T cells on MS progression or symptoms. Finally, the researcher will inspect the search findings to eliminate duplicate studies. The PRISMA-flow diagram for the study is given in Figure 1.

## 2. Results

The keyword combinations yielded 1301 results; plus 20 sources were identified through a regular Google search. The data were then screened for duplicates, and 585 references were eliminated, leaving 736. The results were further checked, eliminating 507 articles whose titles and abstracts lacked conformity to the current research question. The penultimate step involved examining the articles for eligibility by determining if they contained data or findings on MS and T cells; 210 articles were eliminated, leaving only 18 eligible for the study. The PRISMA flowchart below exhibits the above results.

### Study Characteristics

The selected journal articles comprised research conducted globally, with the majority from the United States. The journals comprised clinical trials, cohort studies, randomized double-placebo trials, and retrospective and cross-sectional studies conducted within the past ten years. The prospective studies must delve into the role of T cells in multiple sclerosis and whether they contribute to the worsening of the condition. It is envisaged that the selected articles will provide a clear perspective of the role of T cells in multiple sclerosis.

The studies should also point to the impact of regulating Treg cells during MS management or diagnosis. The studies should either reveal the Expanded Disability Status Scale (EDSS) scores or a finding that the Treg cells regulated the MS progression or prognosis. The above information reveals the extent to which modern therapies use Treg cells to manage MS.

## 3. Discussion

The reviewed literature aligns with previous studies attributing Treg cells to MS diagnosis and therapeutic treatments. Multiple studies reveal how changes in regulatory Treg cells in the body depict the presence of disease. The studies further point to numerous instances where the regulation of Treg cells influenced the progression or relapse among MS patients. Calahorra et al. state that regulatory T cells mediate the adaptability of the immune system through autoimmune responses [6]. The above studies reveal that Treg cell therapies and treatments ensure immune tolerance, suppression of autoimmune responses, adaptation to environmental changes, tuning immune responses, and MS diagnosis. Examining the selected literature through the above perspectives can address whether regulatory T cells can diagnose and aid in monitoring MS progression or relapse compared with the standard treatment protocols or controls.

The research evidence suggests how Treg cells can diagnose MS in healthy patients. Certain biomarkers and immunological factors reveal the presence of MS in patients. Assessing CD4+ or CD8+ counts in the cerebrospinal fluid (CSF) can reveal a patient’s vulnerability to MS. Von Essen et al. found a direct correlation between the prevalence of CD20+ T cells in the CSF and MS severity [7]. Verma et al. also found a substantial correlation between elevated CD25 Tregs and MS severity [3]. Notably, McKinney et al. identified NK8+ killer cells, a CD8+ subset, to possess surrogate markers that indicate relapses in remitting multiple sclerosis [8]. The above findings align with multiple postulations from related research that justify using Treg cells for diagnostics. Sabatino Jr. et al. found that myelin-specific CD8+ T cells triggering CD20 increased among MS patients, revealing the presence of an infection [9]. The finding implies a complex interplay between CD8+ T cells that target myelin and the MS pathogenesis. Kitz et al. also justified that Tregs play a crucial role in MS through genetic changes in FoxP3+ Tregs among MS patients [10]. Kimura argues that the Treg cell’s mechanism of action and MS alteration can point to the existence of a condition [11]. Finally, Canto-Gomes argued that naïve regulatory T cells among patients often symbolize MS [12]. The above studies point to the possibility of using Treg cells in diagnosing MS. Jointly, the findings underscore the multifaceted nature of the MS immune responses, emphasizing the potential diagnostic utility of biomarkers and Treg cells to examine and manage the disease.

The study findings also reveal the theme of Treg cells promoting immune tolerance among affected patients. The immune system can recognize and tolerate self-antigens while attacking foreign elements simultaneously. Visweswaran et al. found that AHSCT therapy induced the recalibration of pro-inflammatory and immunoregulatory components of the immune system, prolonging immune tolerance [13]. On the other hand, Pender observed that Epstein–Barr virus-specific T cell therapy boosted immune tolerance by increasing the potency of the T cells, triggering an immune tolerance [14]. Fitzgerald et al. found that an intermittent CR diet lowered the T cell subsets and certain lipid markers, lowering effector memory for MS markers and improving immune tolerance [15]. Montalban et al. found that administering ocrelizumab reduced MS progression, improving disease symptoms [16]. Finally, Tavaf et al. found that Berberine therapy decreased pro-inflammatory cytokines, relieving the inhibition of Treg cells and promoting immune tolerance [17]. The studies imply that improving the levels of Treg cells, such as CD4+ and CD39+ Tregs, can boost immune tolerance, leading to a relapse in the symptoms. Danikowski et al. affirm the above findings by stating that Tregs maintain peripheral tolerance against antigens through diverse soluble mediators [18]. They release anti-inflammatory cytokines which regulate autoimmune response. Duffy et al. also state that Treg cells are immunosuppressive and regulate the immune system through self-tolerance and by preventing autoimmunity [19]. Sakaguchi et al. state that Treg cells establish immunological unresponsiveness against self-antigens, thereby suppressing immune responses [20]. Traxinger et al. also found that Tregs aid in balancing active immunity against immunological tolerance during hostile antigen exposure [21]. Thus, regulatory Tregs play an active role in immune tolerance by preventing inappropriate or excessive reactions and suppressing the immune responses. The above understanding of the Treg cell function paves the way for diverse therapeutic techniques to improve immune tolerance and prevent autoimmune diseases.

The literature also reveals the ability of the Treg cells to suppress autoimmune responses, thereby influencing MS progression. Multiple studies reveal instances where Treg cells suppressed autoimmune responses, improving the MS symptoms. Multiple studies revealed a reduction in the EDSS scores from the baseline, proving the suppression of the autoimmune responses. Choi et al. found that fasting-mimicking diet therapy increased the T cell level, causing autoimmune cell regeneration [22]. Gilmore et al. observed that repopulating T, B, and NK cells after alemtuzumab therapy caused patients to develop T2 lesions, promoting immune tolerance [23]. Fleming further observed that Trichuris suis (TSO) therapy improved T cell volume, leading to immune tolerance [24]. Finally, Tanasescu et al. found that patients under Hookworm treatment therapy improved the T cell level in patients, thereby suppressing the autoimmune responses [25]. Rajendiran and Tenbrock consider the regulatory T cells as important immune system gatekeepers that express CD4+, CD25+, and FoxP3+ regulatory T cells to inhibit CD4+ helper and cytotoxic CD8+ T cell activation [26]. Goswami et al. also found that human Tregs have subpopulations, such as CD45RA+FoxP3 low resting Tregs and CD45RA-FoxP3 high activated Tregs, which suppress the innate autoimmune responses [27]. Schlöder et al. also observed that the activation of Treg triggers a strong immunosuppressive property, such as the inhibition of the T cell-mediated immune responses against the self-antigens, thereby preventing autoimmunity [28]. Mikami et al. also observed a similar trend where iTreg cells generated under CD28 signal deprivation gained a stable Treg-specific DNA hypomethylation after vivo transfer, thereby suppressing antigen-specific immune responses [29]. Rajendiran and Tenbrock (2021) found that CD4+, CD25+, and FoxP3+ regulatory T cells inhibited CD4 helper cell activation [26]. Eggenhuizen et al. revealed that Tregs can suppress antibody production and autoreactive B cells, lowering autoimmune responses [30]. The above references augment the theme of Treg cell-mediated suppression of autoimmune responses and provide a robust foundation for understanding the diverse mechanisms and subpopulations involved in this crucial immunomodulatory process. The evidence positions Treg cells as integral in shaping the MS autoimmune trajectory, creating avenues for diverse therapeutic interventions to harness the immunosuppressive potential.

The studies also reveal the ability of Treg cells to adapt to environmental changes in response to distinct levels of inflammation. Immune systems can encounter increased inflammation; in such cases, Treg cells are activated to improve suppressive function and regulate immune responses. Glatigny et al. found that Abatacept transcriptional targets triggered CD28-mediated costimulatory signaling, which activated the expression of the desired target genes, revealing the adaptability of Treg cells to specific inflammatory cues [31]. McKinney also observed that the generation of network transcriptomics to CD8+ cells leads to a signature reflecting the expansion of a subset of CD8+ natural killer cells (NK8+) [8]; the above outcome suggests an adaptive modulation of immune responses within the CD8+ cell subset. Iannetta et al. revealed the adaptive nature of immune responses generated by vaccination, specifically in developing memory T cells [32]; the outcome demonstrates the immune system’s capacity to capture and retain information about encountered pathogens. Finally, Cignarella et al. found that intermittent fasting increases the number of regulatory T cells, suggesting that intermittent fasting triggers an immune adaptive response to optimize regulatory T cell functions [33]. Multiple studies also point to the above observation, justifying the adaptation of Treg cells to address inflammation. Van der Veeken et al. found that eukaryotic cells have a memory of transient encounters alongside a broad range of stimuli-inducing states [34]. The memory of transient encounters enables the Tregs to adapt to new conditions, enabling them to manage inflammation. Brown et al. also illustrated the adaptability of Treg cells to genetic and environmental triggers to influence disease progression [35]. For instance, type 1 diabetes stems from a complex interaction between environmental and genetic factors that influence disease pathology, thereby affecting the progression. Sun et al. also observed that Treg cells can adapt to distinct environmental stimuli and subsequently mirror the effectors [36]. Chaudhry and Rudensky attributed Tregs to the control of homeostasis of the immune system by restraining inflammatory responses to allergens, self-antigens, and pathogens [37]. Billroth-MacLurg et al. found that Treg genes in the experiment had inflammation-specific changes [38]. Alvarez et al. also postulate that Treg cells can adapt to changes within the local micro-environment in the body [39]. Niec et al. found that Treg cells have tissue repair and immune suppression capabilities in their functions [40]. The findings indicate that researchers can alter the genetic profiles of Treg cells in response to inflammatory stimuli. The collective evidence emphasizes the adaptability of Treg cells to changes in the environment during infection. The Treg cells can adapt to distinct levels of inflammation, environmental stimuli, and genetic triggers. The cells can adapt to different MS triggers to minimize inflammations.

The findings also point to the role of Treg cells in tuning immune responses by modulating the intensity and duration of the immune responses. Multiple studies in the review reveal the ability of Treg cells to modulate the autoimmune responses. Vucic et al. found that raising the CD4+ T cell count improved neurological functions [41]. The high CD4+ counts optimize neurological functions to acceptable levels. Glatigny et al. also observed that Abatacept therapy optimized T cell levels, triggering the expression of desired target genes to suppress autoimmune disease [31]. Iannetta et al. observed that administering SARS-CoV-2 vaccines triggered T cells responses, which optimized the body’s immune system to fight the COVID virus [32]. The Treg cell tuning affected cytokine production and regulated metabolism, causing adaptive responses and suppressing autoimmune effects, thus enabling the body to overcome autoimmune conditions. A plethora of research affirms the above observations, revealing their influence in regulating MS. Kojima et al. found that bystander Tregs fine-tune highly antigen-specific immune responses [42]. Sambucci et al. also observed the ability of Treg cells to adopt diverse mechanisms to down-modulate immune responses in the lymphoid and non-lymphoid tissues [43]. Verreycken et al. state that the regulatory Treg cells can suppress autoreactive immune cells that accelerate MS pathology [44]. The study findings reveal that Treg therapy can improve the disease symptoms. In a different study, Schroeter et al. observed that Tregs regulate immune capacity by influencing bystander responses such as autoimmune diseases and allergies [45]. Jones and Hawiger also found that T cells enhance the recovery from MS by suppressing autoreactive cells [46]. Howlett-Prieto et al. found that long-term treatment with ocrelizumab enriched CD8+ CD28− regulatory T cells, thereby improving the symptoms [47]. A comprehensive analysis of the above studies depicts regulatory Treg cells as integral in fine-tuning immune responses to modulate autoimmune reactions and in promoting therapeutic interventions to improve recovery of autoimmune conditions such as MS.

Certain studies revealed that the modulation of Tregs had no significant impact on MS progression. The findings imply that the dysregulation of Tregs may not affect the diagnosis or treatment of MS. Rolf et al. found that stimulated CD8+ T cells caused no significant difference in the pro- and anti-inflammatory cytokine balances [48]. Mähler et al. also observed that training failed to increase or decrease the amount of CD39+ and CD31+ Tregs [49,50]. Despite the above outcomes, Van Langelaar affirms the role of T cells in slowing the progression of MS. The distinction in the findings reveals a gap requiring more research. The above results could stem from methodological errors or factual findings. The above finding disapproves of the vast findings that Treg cell regulation affects MS.

### 3.1. Treg Cells and EAE

Experimental autoimmune encephalomyelitis (EAE) studies also depict the role of regulatory T cells in immune system regulation and tolerance. Tregs enforce a control mechanism by suppressing immune responses, preventing the body’s immune system from attacking the tissues. Rossi reveals that EAE studies simulate clinical patterns and histopaths for demyelinating inflammations in the central nervous system [51]. The systematic literature review identified six studies that examine the influence of Treg cells on EAE. The findings demonstrate how the imbalance between T cells and effector T cells influences EAE susceptibility, the role of regulatory T cells in the recovery of EAE, and how CD8+ T cells inhibit autoimmune demyelination.

The imbalance between T cells and effector T cells disrupts the equilibrium for effective immune system regulation, leading to disease progression. Depleting CD4+ and CD25+ regulatory T cells increases the susceptibility of the mice due to decreased immune suppression responses. Ghosh found that the mice that had depleted CD4+ and CD25+ experienced severe symptoms of EAE [52]. Leavenworth et al. also observed that the mice with FoxP3-specific deletion experienced severe EAE [53]. Multiple EAE studies have also exhibited findings that support the above observations. Dong found that effector T cells target cytokines, thereby suppressing the adverse symptoms of immune disorders [54]. Okeke also observed that Tregs regulate inflammatory disease pathogenesis and maintain immune tolerance, raising the body’s ability to suppress autoimmune disorders [55]. An imbalance in the Tregs predisposes the body to autoimmune attacks since the available Tregs may not sufficiently protect against the diseases. The above findings demonstrate the need to balance the T cell population to minimize the onset and progression of EAE.

Regulatory T cells mediate the recovery of EAE by availing ideal cells to suppress autoimmune functions. The Tregs dampen autoimmune responses by controlling the response and functions of the T cell population. Koutrolos et al. found that regulatory T cells control the proliferation and motility of the effector T cells [56]. McIntyre et al. also found that regulatory T cells boosted remyelination after neural stem cell transplant [57]. The above outcomes are consistent with diverse studies that portray similar themes. Hosseinalizadeh et al. also observed that transmitting Tregs mitigates EAE severity, and its withdrawal can exacerbate the condition [58]. Baeten also illustrated the immune dysregulation polyendocrinopathy enteropathy X-linked (IPEX) syndrome, where the FoxP3 gene was mutated to eliminate Tregs entirely. The transfer of Tregs improved the EAE symptoms in the mice [59]. The above findings demonstrate the role of regulatory T cells in ensuring the recovery of EAE among patients with severe symptoms.

The findings also demonstrate the role of Tregs in inhibiting autoimmune demyelination. The Tregs induce diverse mechanisms, such as secreting immunosuppressive cytokines to prevent autoreactive cell activation and prevent pro-inflammatory responses [60]. Choi also discovered that subjecting mice to a diet mimicking fasting increased Treg cell levels, thereby preventing demyelination [22]. The above findings are consistent with study postulations that Tregs promote myelin repair and induce demyelination, inhibiting autoimmune response [61]. Romano also found that regulating Tregs induces peripheral tolerance, inhibiting the autoimmune responses [62]. The results jointly reveal the ability of Tregs to prevent autoimmune diseases.

### 3.2. Clinical Guidelines Using Regulatory T Cells Measurement in MS Patients

The study points to the diverse roles of Tregs in the diagnosis and management of MS and EAE. The above findings infer specific cases when researchers or healthcare practitioners can implement the above functions and roles to mitigate the disease. The human and animal studies provide multiple instances that justify the modulation of Tregs to achieve desirable results. Some of the guidelines for using T cells include research contexts, exploring MS biomarkers, treatment monitoring, and customized treatment modules. Below is an in-depth perspective of the guidelines.

Research on the relationship between Tregs and MS or EAE provides insights into immune system dysregulation among MS patients. The insights enable researchers to understand the correlation between Treg levels and disease activity. For instance, Ciccocioppo observed that increasing the number of Tregs slowed the progression of MS [63]. Such findings enable practitioners to coin effective treatment plans to ensure the systematic regulation of T cells to achieve optimum results. Obtaining exact values on the correlation between Treg levels and disease activities or progression will augment the quality of the interventions.

The correlation between Tregs and MS or EAE could reveal essential biomarkers for the disease. The biomarkers reveal a patient’s immune status, thereby revealing the disease prognosis. Paul et al. state that biomarkers can predict disease progression activity levels, monitor recovery, and examine treatment response [64]. Docampo also points out that reduced, unaltered, or increased Tregs could form strategic biomarkers for disease progression [65]. Identifying specific biomarkers for MS progression in response to Treg therapies can reveal specific biomarkers that offer valuable insights into the disease progression and efficacy of the relevant interventions. Researchers could use the biomarkers to set standards for diagnosing, monitoring recovery, and examining treatment responses for MS patients.

The relationship between Tregs and MS or EAE reveals its significance in treatment monitoring. Healthcare providers can monitor Treg levels to determine the efficacy of the disease therapies. Ma et al. found that differentiating Th17/Treg offers a dynamic balance that prevents inflammatory diseases [66]. Verreycken et al. also observed that MS patients have disturbed Treg numbers, which could reveal a patient’s response to the intervention [44]. Practitioners can set ideal balance thresholds to indicate a hopeless, poor, favorable, moderate, or excellent prognosis. Using Tregs to set the ideal thresholds enhances the accuracy of treatments and choice of therapy.

The knowledge of the influence of Tregs on MS could also enable practitioners to customize a treatment plan for a patient. Customized treatment plans require information about an individual’s immune profile and Treg level measurements. Buc et al. found that the Treg varieties could reveal the levels of adhesive and costimulatory cells, enabling healthcare practitioners to propose ideal interventions [67]. Khosravi also observed a significant correlation between the frequency of Tregs and the EDSS scores [68]. Pohl also indicates that understanding distinct polyclonal regulatory T cells offers diverse treatment options depending on the variants affecting a patient. Examining the relevant treatment parameters offers a blueprint for customizing treatment that will quickly alleviate MS symptoms [69]. Table 1 gives an overview about the most relevant studies in the field and about their characteristics.

## 4. Conclusions

The review solidifies the integral role of regulatory Treg cells in distinct dimensions of MS or EAE, commencing from the diagnostic potential to diverse therapeutic interventions. The selected empirical literature, alongside other supporting evidence, affirms the diagnostic capacity of Treg cells through immunological elements and biomarkers, providing valuable insights into its role in managing MS. The reviewed literature also depicts Treg cells as integral in promoting immune tolerance, adapting to new environments, suppressing autoimmune responses, and tuning immune responses. The changes in the EDSS scores reveal the impacts of Treg cells in the regulation and modulation of MS. Whereas two out of eighteen studies found no correlation between Treg cell regulation and MS progression, most of the articles affirmed the relationship, justifying the notion that Treg cell regulation affects MS. The PICO question, through the research findings, presents a critical aspect of future research, paving the way for insights into the significance of regulatory T cells in the diagnosis and therapy monitoring of patients. The findings can trigger a paradigm shift in treatment from traditional interventions to effective Treg cell therapies.

## Figures and Tables

**Table 1:** Quality Assessment Table.

Study Identification	Study Design	Research Subjects	Number of MS Cases	Number of Healthy Controls	Regulatory T Cell Definition	MS Sclerosis Duration	EDSS Criteria Reported by Authors	EDSS Score	Mean Age of MS	Mean Age in Healthy Controls	Conclusion	Source
Verma, N. D., Lam, A. D., Chiu, C., Tran, G. T., Hall, B. M., & Hodgkinson, S. J. (2021). Multiple sclerosis patients have reduced resting and increased activated CD4+ CD25+ FOXP3+ T regulatory cells. *Scientific Reports*, *11*(1), 10476.	Cohort Studies	Human Subjects	N = 36	N = 20	CD4+ T	8.9 (0.1–25.2) Years	McDonald Criteria 2017	Baseline (MS) = 3.71 After Treatment = 3.04 No Treatment = 4.21	MS = 42.5 (21–64)	HD = 41.7 (21–69)	The study found an increased proportion of CD25 Treg in most MS patients.	[3]
Choi, I. Y., Piccio, L., Childress, P., Bollman, B., Ghosh, A., Brandhorst, S., … & Longo, V. D. (2016). A diet mimicking fasting promotes regeneration and reduces autoimmunity and multiple sclerosis symptoms. *Cell Reports*, *15*(10), 2136–2146.	Experimental Trial	EAE (Mice)	Experimental autoimmune encephalomyelitis (EAE) N = 23 Therapeutic FMD cycles (FMD(T); N = 23)	EAE CTRL N = 23 Ketogenic diet (EAE KD); N = 13 Semi-therapeutic FMD cycles (EAE FMD(S); N = 7	CD4+ or CD8+ T cells in the spinal cord	6 Months	Not Stated	CD (Baseline) = 0 (0 to 0) After Treatment = 0 (0 to 0.5) FMD (Baseline) = 0 (−1 to 0) After Treatment = 0 (−0.5 to 0.1) KD (Baseline) = 0 (−0.5 to 0) After Treatment = 0 (−0.5 to 0) (*p* < 0.05) 3	Not Indicated	Not Indicated	The fasting-mimicking diet (FMD) increased Treg cell level, causing autoimmune cells to regenerate.	[22]
Gilmore, W., Lund, B. T., Li, P., Levy, A. M., Kelland, E. E., Akbari, O., … & Traboulsee, A. L. (2020). Repopulation of T, B, and NK cells following alemtuzumab treatment in relapsing-remitting multiple sclerosis. *Journal of Neuroinflammation*, *17*(1), 1–21.	Clinical Trial	Human Subjects	N = 28	None	CD4+ CD8+	48 Months	McDonald Criteria	Baseline = 5.5 After Treatment = 2.2	Not Indicated	Not Indicated	The study found that 11 patients developed new T2 lesions while 8 had relapses. The findings point to a positive impact in decreasing levels of T cells to treat MS.	[23]
Fleming, J., Hernandez, G., Hartman, L., Maksimovic, J., Nace, S., Lawler, B., … & Fabry, Z. (2019). Safety and efficacy of helminth treatment in relapsing-remitting multiple sclerosis: Results of the HINT 2 clinical trial. *Multiple Sclerosis Journal*, *25*(1), 81–91.	Clinical Trial	Human Subjects	N = 16	None	CD25+, CD127−, CD4+	14 Months	McDonald Criteria	Baseline = 1.3 ± 0.9 Last Treatment = 1.1 ± 1.3	32 (±8)	32	The Trichuris suis (TSO) proved effective in relapsing MS symptoms, and the T cell volume significantly improved.	[24]
Tanasescu, R., Tench, C. R., Constantinescu, C. S., Telford, G., Singh, S., Frakich, N., … & Pritchard, D. I. (2020). Hookworm treatment for relapsing multiple sclerosis: A randomised double-blinded placebo-controlled trial. *JAMA Neurology*, *77*(9), 1089–1098.	Randomized Double-Blinded Placebo-Controlled Trial	Human Subjects	N = 35	N = 36	CD4+	36 Weeks	McDonald Criteria	Placebo 3 (1.5–5) Hookworm 3 (1.5–5)	45	36	The group undergoing the Hookworm treatment experienced relief in symptoms and a subsequent increase in T cells.	[25]
Mähler, A., Balogh, A., Csizmadia, I., Klug, L., Kleinewietfeld, M., Steiniger, J., … & Paul, F. (2018). Metabolic, mental and immunological effects of normoxic and hypoxic training in multiple sclerosis patients: A pilot study. *Frontiers in Immunology*, *9*, 2819.	Randomized Single-Blinded Parallel-Group Study	Human Subjects	N = 34	N = 16	CD4+ CD31+	4 Weeks	McDonald Criteria	Baseline = <4.5	40	40	The training did not increase or decrease the amount of CD39+ and CD31+ Tregs. Therefore, the T cells could not account for the increase in erythropoietin.	[49]
Glatigny, S., Höllbacher, B., Motley, S. J., Tan, C., Hundhausen, C., Buckner, J. H., … & Bettelli, E. (2019). Abatacept targets T follicular helper and regulatory T cells, disrupting molecular pathways that regulate their proliferation and maintenance. *The Journal of Immunology*, *202*(5), 1373–1382.	Double-Blinded Placebo-Controlled Trial	Human Subjects	N = 65	N = 19	CD4+	52 Weeks	Not Stated	Not Indicated	Not Indicated	Not Indicated	The Abatacept treatment showed a selective decrease in CD4+ T follicular helper (Tfh) and regulatory T cells, revealing its inability to support sustained tolerance. However, the generation of the T cells improved the patient’s symptoms.	[31]
McKinney, E. F., Cuthbertson, I., Harris, K. M., Smilek, D. E., Connor, C., Manferrari, G., … & Smith, K. G. (2021). A CD8+ NK cell transcriptomic signature associated with clinical outcome in relapsing-remitting multiple sclerosis. *Nature Communications*, *12*(1), 635.	Clinical Trial	Human Subjects	N = 79	N = 225	CD4+ CD8+	18 Months	Not Stated	Not Indicated	Not Indicated	Not Indicated	The findings revealed that NK8+ killer cells, a subset of CD8+, have surrogate markers that indicate a relapse in remitting multiple sclerosis.	[8]
Iannetta, M., Landi, D., Cola, G., Campogiani, L., Malagnino, V., Teti, E., Coppola, L., Di Lorenzo, A., Fraboni, D., Buccisano, F., Grelli, S., Mozzani, M., Zingaropoli, M. A., Ciardi, M. R., Nisini, R., Bernardini, S., Andreoni, M., Marfia, G. A., & Sarmati, L. (2022). B- and T-Cell responses after SARS-CoV-2 vaccination in patients with multiple sclerosis receiving disease-modifying therapies: Immunological patterns and clinical implications. *Frontiers in Immunology*, *12*, 796482.	Clinical Trial	Human Subjects	N = 40	N = 30	CD4+ or CD8+ T cells in the spinal cord	6 Months	McDonald Criteria	Baseline = 2 (0–3.0) Ocrelizumab (OCR) = 2 (2.0–4.5) Fingolimod (FTY) = 1.5 (1.0–3.0) Natalizumab (NAT) = 0.5 (0.0–2.5)	Not Indicated	Not Indicated	The SARS-CoV-2 vaccination triggered T cell responses, leading to improved symptoms.	[32]
Rolf, L., Muris, A. H., Bol, Y., Damoiseaux, J., Smolders, J., & Hupperts, R. (2017). Vitamin D_3_ supplementation in multiple sclerosis: Symptoms and biomarkers of depression. *Journal of the Neurological Sciences*, *378*, 30–35.	Randomized Pilot Study	Human Subjects	N = 20	N = 20	CD8+ T cells	48 Weeks		Placebo = 2.0 (1.5–2.3) Vitamin D3 = 2.0 (1.5–2.5)	Vitamin D3 = 38.5	Placebo 37.6	The study found that the stimulated CD8+ T cells caused no significant differences in pro- and anti-inflammatory cytokine balances.	[48]
Von Essen, M. R., Ammitzbøll, C., Hansen, R. H., Petersen, E. R. S., McWilliam, O., Marquart, H. V., Damm, P., & Sellebjerg, F. (2019). Proinflammatory CD20+ T cells In the pathogenesis of multiple sclerosis. *Brain: A Journal of Neurology*, *142*(1), 120–132.	Clinical Trial	Human Subjects	N = 25	N = 25	CD3 CD4 CD20+	2 Years	McDonald Criteria 2010	CDSS Improved from the Baseline	37	36	The findings showed an increased amount of CD20+ T cells in the blood of MS patients.	[7]
Vucic, S., Ryder, J., Mekhael, L., Henderson, R. D., Mathers, S., Needham, M., … & Kiernan, M. C. (2020). Phase 2 randomized placebo-controlled double-blind study to assess the efficacy and safety of tecfidera in patients with amyotrophic lateral sclerosis (TEALS Study): Study protocol clinical trial (SPIRIT Compliant). *Medicine*, *99*(6).	Randomized Placebo-Controlled Double-Blinded Trial	Human Subjects	N = 60	N = 30	CD4+ T cells CD45RO+	40 Weeks	Not Stated	Not Indicated	Not Indicated	Not Indicated	The findings reveal that increasing CD4+ T cell count improves the body’s neurological functions and prolongs the patient’s survival.	[41]
Cignarella, F., Cantoni, C., Ghezzi, L., Salter, A., Dorsett, Y., Chen, L., … & Piccio, L. (2018). Intermittent fasting confers protection in CNS autoimmunity by altering the gut microbiota. *Cell Metabolism*, *27*(6), 1222–1235.	Randomized Controlled Trial	Human Subjects	N = 17	N = 10	CD4+ T cells	4 Weeks	McDonald Criteria	AD Libitum (Baseline) = 3.7 (2.7–5.2) Intermittent Fasting (Baseline) = 3.7 (3.2–4)	40 ± 12	42 ± 8.2	The findings revealed that intermittent fasting (IF) increased the number of regulatory T cells, improving the MS symptoms.	[33]
Tavaf, M. J., Soltanmohammadi, A., Zargarani, S., Yazdanpanah, E., Sadighimoghaddam, B., Yousefi, B., … & Haghmorad, D. (2023). Berberine promotes immunological outcomes and decreases neuroinflammation in the experimental model of multiple sclerosis through the expansion of Treg and Th2 cells. *Immunity, Inflammation and Disease*, *11*(1), e766.	Experimental Trial	Mice	6 Mice 2 Mice—Low Dose Berberine 2 Mice—High Dose Berberine	2 Mice Control	CD4+ T cells	25 Days	Not Stated	Not Indicated	8–10 Weeks	8–10 Weeks	The treatment groups had decreased pro-inflammatory cytokines, which relieved the inhibition of Treg cells and hence improved symptoms.	[17]
Montalban, X., Hauser, S. L., Kappos, L., Arnold, D. L., Bar-Or, A., Comi, G., de Seze, J., Giovannoni, G., Hartung, H. P., Hemmer, B., Lublin, F., Rammohan, K. W., Selmaj, K., Traboulsee, A., Sauter, A., Masterman, D., Fontoura, P., Belachew, S., Garren, H., Mairon, N., … ORATORIO Clinical Investigators (2017). Ocrelizumab versus placebo in primary progressive multiple sclerosis. *The New England Journal of Medicine,* 376(3), 209–220.	Randomized Placebo-Controlled Trial	Human Subjects	N = 732	N = 244	CD3+ CD4+ CD8+ cells	12 Weeks	McDonald Criteria	Baseline = 3.0–6.5 After Treatment = 1.5 (1.0–3.0)	43.2	40.1	The infusion of ocrelizumab was associated with reduced clinical disease progression compared with the placebo group.	[16]
Pender, M. P., Csurhes, P. A., Smith, C., Douglas, N. L., Neller, M. A., Matthews, K. K., Beagley, L., Rehan, S., Crooks, P., Hopkins, T. J., Blum, S., Green, K. A., Ioannides, Z. A., Swayne, A., Aftab, B. T., Hooper, K. D., Burrows, S. R., Thompson, K. M., Coulthard, A., & Khanna, R. (2018). Epstein–Barr virus-specific T cell therapy for progressive multiple sclerosis. *JCI Insight*, *3*(22), e124714.	Clinical Trial	Human Subjects	N = 13	N = 13	CD4+ CD8+ T cells	27 Weeks	Revised McDonald Criteria	Baseline = 8.0 After Treatment ≤ 6.5	Not Indicated	Not Indicated	The EBV-specific T cell therapy reduced the EDSS score, preventing further autoimmune attacks.	[14]
Fitzgerald, K. C., Bhargava, P., Smith, M. D., Vizthum, D., Henry-Barron, B., Kornberg, M. D., Cassard, S. D., Kapogiannis, D., Sullivan, P., Baer, D. J., Calabresi, P. A., & Mowry, E. M. (2022). Intermittent calorie restriction alters T cell subsets and metabolic markers in people with multiple sclerosis. *eBioMedicine*, *82*, 104124.	Randomised Controlled Feeding Study	Human Subjects	N = 36	N = 12	CD4+ CD+ T cells	8 Weeks	McDonald Criteria	Baseline < 6.5 Intermittent CR = 1.75 (0.72) Daily CR = 1.67 (0.91) Control CR = 1.08 (1.14)	37.4	37.4	The findings show that an intermittent CR diet reduced T cell subsets and specific biologically relevant lipid markers, causing a significant reduction in the effector memory for MS markers.	[15]
Visweswaran, M., Hendrawan, K., Massey, J. C., Khoo, M. L., Ford, C. D., Zaunders, J. J., Withers, B., Sutton, I. J., Ma, D. D. F., & Moore, J. J. (2022). Sustained immunotolerance in multiple sclerosis after stem cell transplant. *Annals of Clinical and Translational Neurology*, *9*(2), 206–220.	Randomized Clinical Trial	Human Subjects	N = 22	N = 18	CD4+ CD8+ CD57+ T cells	36 Months	McDonald Criteria	Baseline = 7.0 After Treatment = 2.0	34	34.5	The study findings revealed that the Autologous Hematopoietic Stem Cell Transplantation increased the CD4+ Tregs and CD39+ Treg percentages, lowering disease symptoms.	[13]
Koutrolos M, Berer K, Kawakami N, Wekerle H, Krishnamoorthy G. Treg cells mediate recovery from EAE by controlling effector T cell proliferation and motility in the CNS. Acta neuropathologica communications. 2014 Dec;2:1–7.	Experimental Trial	EAE (DEREG Mice)	N = 11	N = 5	CD45+ CD4+ FoxP3− T cells	6 Days	Not Stated	Not Indicated	Not Indicated	Not Indicated	The study findings showed that the absence of regulatory T cells decreased the velocity of effector T cells. The study concludes that regulatory T cells mediate recovery from EAE.	[56]
Leavenworth JW, Luo L, Hu X, Dixon ML, Pope BJ, Leavenworth JD, Raman C, Meador WR. Dysregulated follicular regulatory T cells and antibody responses exacerbate CNS autoimmunity. The Journal of Immunology. 2021 May 1;206(1_Supplement):51–11.	Experimental Trial	EAE (Mice)	N = 5	N = 5	CD103 (C) CD69 (D) FoxP3+ Tregs	20 Days	Not Stated	Not Indicated	7 Weeks	7 Weeks	The study revealed that mice with FoxP3-specific deletion of Blimp1 developed severe EAE and did not recover compared with the mice in the control group.	[53]
McIntyre LL, Greilach SA, Othy S, Sears-Kraxberger I, Wi B, Ayala-Angulo J, Vu E, Pham Q, Silva J, Dang K, Rezk F. Regulatory T cells promote remyelination in the murine experimental autoimmune encephalomyelitis model of multiple sclerosis following human neural stem cell transplant. Neurobiology of disease. 2020 Jul 1;140:104868.	Experimental Studies	EAE (Mice)	eGFP-mNSCs (N = 12)	PBS (N = 13)	CD4+ CD25+ FoxP3+ regulatory T cells	21 Days	Not Stated	Not Indicated	8 Weeks	8 Weeks	The study showed that eight weeks of mice receiving hNSCs at the chronic stage experienced reduced neuroinflammation, remyelination, and an increase in CD4+, CD25+, FoxP3+ regulatory T cells.	[57]
Kashi VP, Ortega SB, Karandikar NJ. Neuroantigen-specific autoregulatory CD8+ T cells inhibit autoimmune demyelination through modulation of dendritic cell function. Plos one. 2014 Aug 21;9(8): e105763.	Experimental Studies	EAE (Mice)	N = 15	N = 15	CD8+ CD4+ T cells	12 Days	Not Stated	Not Indicated	7 Weeks	7 Weeks	The study found that CD8+ T cells inhibit autoimmune demyelination in EAE, relieving the disease symptoms.	[60]
Ghosh, D., Curtis, A. D., Wilkinson, D. S., & Mannie, M. D. (2016). Depletion of CD4+ CD25+ regulatory T cells confers susceptibility to experimental autoimmune encephalomyelitis (EAE) in GM-CSF-deficient *csf2*−/− mice. *Journal of Leukocyte Biology*, *100*(4), 747–760. https://doi.org/10.1189/jlb.3a0815-359r	Experimental Studies	EAE (Mice)	N = 18	N = 19	e CD4+ CD25+ FoxP3+ T cell	41 Days	Not Stated	Not Indicated	Not Indicated	Not Indicated	The study findings show that Csf2-deficient mice resisted EAE due to the imbalance between T cells and effector T cells.	[52]

## Data Availability

Not applicable.
